# Association of Salivary HIF‐1α Level With Epithelial and Mesenchymal Markers in Smokers and Nonsmokers With Periodontitis: A Case‐Control Study

**DOI:** 10.1155/ijod/2103392

**Published:** 2026-08-26

**Authors:** Ban Emad Kareem, Saif Sehaam Saliem

**Affiliations:** ^1^ Department of Periodontics, College of Dentistry, University of Baghdad, Baghdad, Iraq, uobaghdad.edu.iq

**Keywords:** E-cadherin, hypoxia-inducible factor-1α, N-cadherin, periodontitis, smoking

## Abstract

**Objectives:**

This case–control study aimed to investigate the relationship between the hypoxia‐related biomarker hypoxia‐inducible factor‐1α (HIF‐1α) and the epithelial‐mesenchymal transition (EMT)–associated markers E‐cadherin (E‐CAD) and N‐cadherin (N‐CAD) in saliva among smokers and nonsmokers with and without periodontitis and to assess their association with clinical periodontal parameters.

**Methods:**

A case–control study was conducted, including 88 participants equally allocated into four groups (*n* = 22 each): healthy nonsmokers, healthy smokers, nonsmokers with periodontitis, and smokers with periodontitis. Unstimulated whole saliva samples were collected, and periodontal status was assessed using plaque index (PI), bleeding on probing (BOP), probing pocket depth (PPD), and clinical attachment loss (CAL). Subsequently, samples were analyzed for HIF‐1a, E‐CAD, and N‐CAD levels using enzyme‐linked immunosorbent assay (ELISA). Statistical analyses included intergroup comparisons, Spearman’s correlation analysis, diagnostic performance evaluation using receiver operating characteristic (ROC) curve analysis, and mixed‐effects model analysis.

**Results:**

Salivary E‐CAD and N‐CAD levels were significantly higher in the periodontitis groups than in the healthy groups (*p*  < 0.05), whereas HIF‐1α showed no significant differences among the study groups (*p*  > 0.05). All clinical periodontal parameters were significantly elevated in participants with periodontitis. ROC analysis demonstrated acceptable discriminatory performance for E‐CAD and N‐CAD, whereas HIF‐1α showed variable performance. N‐CAD yielded the highest discrimination between periodontal health and periodontitis (area under the curve [AUC] = 84.5%), while E‐CAD showed the highest discrimination between healthy smokers and nonsmoking participants with periodontitis (AUC = 85.9%). Although salivary E‐CAD levels were descriptively lower in smokers with periodontitis than in nonsmokers with periodontitis, this difference was not statistically significant.

**Conclusions:**

Salivary E‐CAD and N‐CAD were significantly altered in periodontitis and demonstrated greater potential than HIF‐1α for reflecting periodontal disease status. The absence of significant differences in salivary HIF‐1α levels among the study groups may indicate limited ability to reflect localized periodontal hypoxic changes. HIF‐1α also showed limited and inconsistent associations with EMT‐related biomarkers, with a significant positive correlation observed only between HIF‐1α and N‐CAD in the periodontitis group. Smoking was associated with alterations in biomarker profiles, particularly E‐CAD levels. Overall, salivary EMT–related biomarkers may serve as promising noninvasive indicators of periodontal disease, although further validation is required.

## 1. Introduction

Periodontitis is a complex inflammatory condition that facilitates the chronic degradation of the tooth’s functional apparatus as a consequence of host‐mediated inflammation in response to dysbiotic plaque [1]. This pathological disorder leads to the apical migration of the junctional epithelium and the deleterious remodeling of alveolar bone compromising periodontal integrity [2]. Smoking and systemic diseases are known to modulate the host‐microbial interactions along with genetic variables, environmental exposures, and acquired disorders [3].

Through the biological process termed epithelial‐mesenchymal transition (EMT), stable epithelial cells obtain unique mesenchymal features that enhance migratory and invasive capabilities [4]. This phenotypic alteration is facilitated by a highly coordinated sequence of cellular changes. EMT specifically involves the disintegration of intercellular adhesions and the breakdown of apical‐basal polarization coupled with significant cytoskeletal reorganization that defines a new anterior–posterior axis [5]. This process is regulated at the molecular level by the reciprocal downregulation of epithelial markers such as E‐cadherin (E‐CAD) and catenin and the upregulation of mesenchymal genes and proteins including Snail, N‐cadherin (N‐CAD), and vimentin [4, 6]. Although EMT is traditionally associated with embryonic development, cancer progression, and fibrosis, it is now widely recognized as a consequence of periodontal infection, particularly by Gram‐negative species [7]. The accompanying degradation of intercellular junctions impairs epithelial barrier function, facilitating microbes’ unrestricted access to the subjacent connective tissues, precipitating local inflammation and tissue destruction [8].

It is widely documented that periodontal deterioration is mainly driven by a recognized consensus of contributing variables, notably the colonization of Gram‐negative pathogens, the upregulation of inflammatory mediators, reduced tissue oxygenation, cigarette use, and redox imbalances [9]. Although the existence of bacterial biofilm is the primary cause of periodontal disease, smoking is considered a critical modifier in the 2018 World Workshop classification of periodontal diseases, emphasizing its role in disease progression and biomarker variability [2]. Numerous epidemiological findings consistently show that smokers have higher rates and greater severity of periodontitis than nonsmokers [10]. Nicotine‐induced vasoconstriction is considered a key mechanism underlying this association, as it reduces periodontal blood flow and compromises the delivery of oxygen and nutrients to the tissues [11, 12]. These vascular alterations may contribute to dysregulated inflammatory responses and the development of localized hypoxia [13, 14]. According to recent research, cigarette smoke may promote EMT in bronchial epithelial cells of the lung through multiple interconnected pathways, including hypoxia, oxidative stress, and inflammatory signaling [15].

Hypoxia is considered an important pathogenic factor in the development and progression of periodontitis [16–18]. Reduced oxygen tension within periodontal pockets favors the proliferation of anaerobic pathogens and intensifies inflammatory responses [19]. The adaptive response to oxygen deficiency in inflamed tissue involves the accumulation and activation of the hypoxia‐inducible factor‐1α (HIF‐1α), a principal transcriptional regulator of cellular adaptation to hypoxia [20, 21]. Evidence from experimental models indicates that enhancing HIF‐1α signaling may facilitate bone formation, limit inflammatory‐cell accumulation, and improve tissue repair [22–24]. Wang et al. [25] demonstrated that hypoxia‐driven HIF‐1α signaling has been linked to EMT‐related pathways. However, whether these pathways are reflected in whole‐saliva biomarker profiles in periodontitis, particularly among smokers, remains insufficiently understood.

Salivary diagnostic techniques have gained considerable attention as simple, noninvasive tools. Patients and other people with some training can collect whole saliva [26]. Salivary biomarkers are measurable molecules that can be used to monitor health status and carry out surveillance of diseases [27]. Therefore, the present study aimed to investigate the association of salivary HIF‐1α levels with epithelial and mesenchymal markers in smokers and nonsmokers with and without periodontitis and to explore their correlation with clinical periodontal parameters.

## 2. Materials and Methods

### 2.1. Study Design and Ethical Considerations

This research was designed as an observational case–control study framework conducted between November 2025 and March 2026. Participants were recruited and clinically examined at the Department of Periodontics, College of Dentistry, University of Babylon. A sample of 88 subjects was included and received a full periodontal examination. For further biochemical analysis, unstimulated saliva specimens were collected from each participant. The research received ethical approval from the Ethics Committee of Clinical Research of the College of Dentistry, University of Baghdad (Approval Number 1119; 27 November 2025). The research protocol conformed to the ethical principles outlined in the Declaration of Helsinki, and all participants were asked to write an informed consent before their inclusion. The Strengthening the Reporting of Observational Studies in Epidemiology (STROBE) guidelines were followed in the evaluation and verification of the study.

### 2.2. Study Population

Participants aged 18 years or older, systemically healthy, had a minimum of 20 permanent teeth (excluding third molars), and without a history of periodontal therapy or drug usage in the preceding 3 months were included.

The exclusion criteria included individuals with systemic disorders or those who had taken antibiotics, anti‐inflammatory drugs, or corticosteroids within the previous 3 months. Pregnant or lactating individuals; those undergoing orthodontic treatment; chemotherapy, or radiotherapy; those taking immunosuppressive drugs; and those who refused to participate were also excluded.

### 2.3. Sample Size Determination and Grouping

An initial pilot assessment was conducted to estimate the required sample size, using salivary E‐CAD concentrations measured by enzyme‐linked immunosorbent assay (ELISA). E‐CAD was selected as the primary biomarker outcome for sample size estimation. According to Sharma et al. [28], the following formula for case–control studies was used to estimate the sample size.

“Sample size = r+1222/r×SD×Zβ+Zα/2/d”.

The total study population comprised 88 participants, who were allocated into four equal groups (*n* = 22 per group) according to periodontal condition and smoking status:

• Healthy nonsmokers (H).
• Healthy smokers (HS).
• Nonsmokers with periodontitis (P).
• Smokers with periodontitis (PS).

A total of 44 participants with periodontitis were included as cases, while 44 periodontally healthy participants served as controls, giving a case‐to‐control ratio of 1:1.

Smoking status was defined as a history of daily cigarette consumption of ≥10 cigarettes for a minimum duration of 5 years. A probing pocket depth (PPD) of ≤3 mm, bleeding on probing (BOP) in less than 10% of sites, and no evidence of clinical attachment loss (CAL) were defined as clinical periodontal health [29]. In accordance with the 2018 classification criteria, the diagnosis of periodontitis was established, requiring the presence of CAL at two or more nonadjacent teeth, the presence of buccal or oral CAL over 3 mm, and PPD over 3 mm, detectable at least two teeth. All individuals in the disease group exhibited a generalized manifestation of the condition (≥30% of teeth affected) and an unstable disease status (PPD 4 mm with BOP or PPD ≥ 5 mm) [30].

### 2.4. Clinical Examination

A standard protocol for full‐mouth periodontal evaluation for all participants was carried out by a trained and calibrated examiner using a UNC‐15 periodontal probe (Hu Friedy, Chicago, USA). With the exclusion of third molars from the evaluation, clinical indices were obtained from six sites per tooth. The indices recorded included plaque index (PI) [31], BOP [32], PPD, and CAL. Subsequent to gentle probing, BOP was noted within a 10‐s interval with a controlled probing force of 20–25 g. The distance from the gingival margin to the base of the periodontal pocket defined the PPD, whereas CAL was measured from the cementoenamel junction to the pocket base. Before the initiation of data collection, examiner calibration was established in collaboration with a periodontics specialist. To ensure measurement reliability, repeated assessments were performed with a 2‐h interval to reduce the likelihood of false‐positive outcomes associated with immediate re‐examination.

### 2.5. Saliva Collection and Biomarker Analysis

Unstimulated saliva was collected from all participants between 9:00 AM and 12:00 PM, in advance to the clinical examination. To minimize variability, participants were asked to avoid food and drink intake, except for water, for at least 1 h before collection. Within a 5‐min period, saliva samples were collected using the passive drool method [33]. Approximately 500 µL of saliva was then transferred into Eppendorf tubes preloaded with 50 µL of protease inhibitor. The samples were subsequently centrifuged at 4000 rpm for 15 min to eliminate debris. The extracted supernatant was carefully isolated and kept at −20°C until further analysis. The concentrations of salivary E‐CAD, N‐CAD, and HIF‐1α were determined using commercially available ELISA kits (Elabscience, USA), following the manufacturer’s specified guidelines.

### 2.6. Statistical Analysis

Statistical procedures were implemented using GraphPad Prism (version 9.0). The normality of the data was evaluated with Shapiro–Wilk test, which revealed a non‐normal distribution for most of the variables except for age. Accordingly, continuous variables were summarized either by mean and standard deviation (SD) or by median and interquartile range (IQR), depending on the distribution pattern. Age comparisons were performed by analysis of variance (ANOVA). Sex distribution among the study groups was compared using the chi‐square test. The Kruskal–Wallis test was used for comparison among the study groups (nonparametric approach), with a subsequent Dunn’s test with Bonferroni correction applied for pairwise comparison (adjusted *p*‐values  < 0.0083). Associations between salivary biomarkers and clinical periodontal parameters were explored using Spearman’s rank–order correlation coefficient. To explore diagnostic performance for the biomarkers, receiver operating characteristic (ROC) curve analysis was performed, with calculation of the area under the curve (AUC) and corresponding optimal cutoff points, sensitivity, and specificity. Additionally, mixed‐effects models were employed to evaluate group‐related effects while accounting for within‐group variability, using the healthy smoker group as the reference category. Statistical significance was defined at *p*  < 0.05.

## 3. Results

### 3.1. Demographic Findings

**Figure 1:**
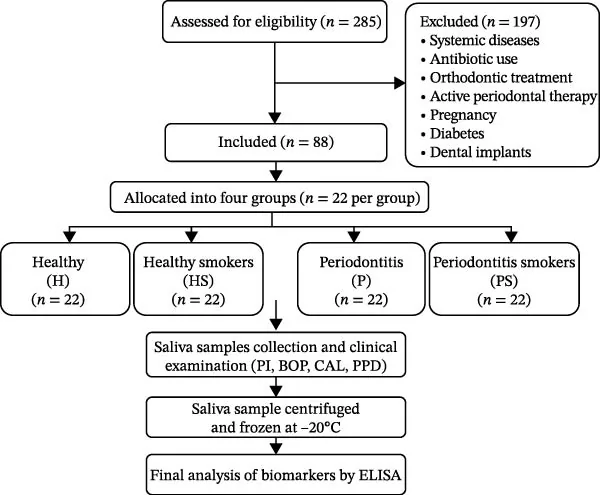
Flow diagram illustrating participant selection, group allocation, clinical examination, and salivary sample processing.

A total of 285 individuals were screened, of whom 197 were excluded according to the predefined inclusion and exclusion criteria. The final sample comprised 88 participants equally allocated into four groups (*n* = 22 each), as shown in Figure 1. The age and sex distribution of the participants are presented in Table 1. No statistically significant difference in mean age was observed among the study groups (*p* = 0.061). However, the lowest mean age was recorded in the healthy group (27.14 ± 9.25 years), whereas the highest was observed in the periodontitis group (40.06 ± 9.59 years). Sex distribution differed significantly among the groups (*p*  < 0.001), with males predominating in the smoking groups.

**Table 1:** Descriptive statistics of demographic and clinical periodontal characteristics of the study groups.

Total (*n* = 88)	H (*n* = 22)	HS (*n* = 22)	P (*n* = 22)	PS (*n* = 22)	*p*‐Value
Age^a^					
Mean ± SD	27.140 ± 9.250	31.010 ± 11.900	40.060 ± 9.590	38.030 ± 10.700	0.061^NS^
Gender (*N*%)^b^					
Male	5 (22.73%)	22 (100%)	13 (59.09%)	21 (95.45%)	<0.001 ^∗∗∗^
Female	17 (77.27%)	0 (0.00%)	9 (40.91%)	1 (4.55%)	
Parameters^c^					
PI%					
Mean ± SD	7.680 ± 1.81	7.590 ± 1.470	66.260 ± 16.250	67.430 ± 18.540	<0.001 ^∗∗∗^
Median	8.030	7.770	65.360	62.700	
BOP%					
Mean ± SD	3.710 ± 2.090	4.170 ± 1.490	51.001 ± 24.140	43.030 ± 11.410	<0.001 ^∗∗∗^
Median	2.970	4.160	47.020	40.470	
PPD (mm)					
Mean ± SD	1.860 ± 0.770	1.820 ± 0.850	4.530 ± 0.430	4.620 ± 0.290	<0.001 ^∗∗∗^
Median	2.00	2.00	4.35	4.65	
CAL (mm)					
Mean ± SD	1.400 ± 0.510	1.500 ± 0.520	2.780 ± 0.670	2.810 ± 0.600	<0.001 ^∗∗∗^
Median	1.000	1.500	2.770	2.760	

Abbreviations: BOP, bleeding on probing; CAL, clinical attachment loss; H, healthy nonsmokers; HS, healthy smokers; N, number; P, nonsmokers with periodontitis; PI, plaque index; PPD, probing pocket depth; PS, periodontitis smokers; SD, Standard deviation.

^a^Comparison using one‐way ANOVA.

^b^Gender distribution was performed using chi‐square test.

^c^Comparison using the Kruskal–Wallis test.

^∗∗∗^ *p* < 0.001 statistically significant.

### 3.2. Clinical Findings

**Figure 2:**
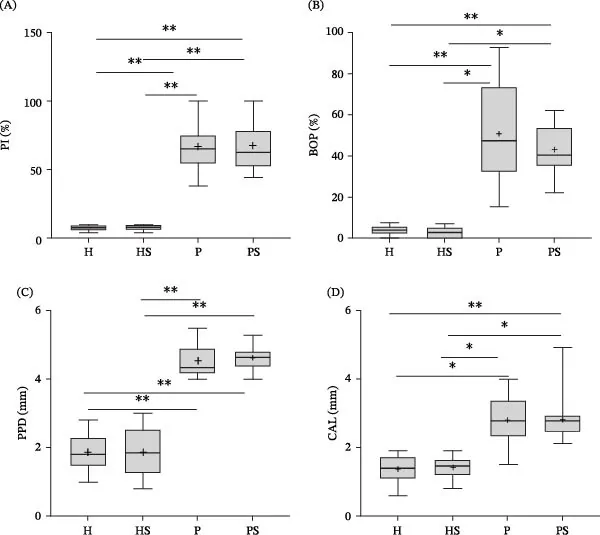
Comparison of clinical periodontal parameters across study groups. (A) Plaque index (PI), (B) bleeding on probing (BOP), (C) probing pocket depth (PPD), and (D) clinical attachment loss (CAL) among healthy nonsmokers (H), healthy smokers (HS), periodontitis nonsmokers (P), and periodontitis smokers (PS). Data are presented as median by the bisecting lines of boxes, with error bars indicating minimum and maximum values. Crosses within boxes represent mean values. Intergroup comparisons were performed using the Kruskal–Wallis test followed by Dunn’s post hoc test with Bonferroni correction. Statistically significant differences are indicated as  ^∗^ *p*  < 0.05 and  ^∗∗^ *p*  < 0.01.

All clinical periodontal parameters including PI, BOP, PPD, and CAL differed significantly among the study groups (*p*  < 0.001). Participants with periodontitis (P and PS) showed significantly higher values for all parameters than healthy participants (H and HS). No significant differences were observed between H and HS or between P and PS in post hoc comparisons (*p*  > 0.05). Although smokers with periodontitis showed descriptively higher values than nonsmokers with periodontitis, these differences did not reach statistical significance (Table 1 and Figure 2).

### 3.3. Salivary Levels of E‐CAD, N‐CAD, and HIF‐1α

**Figure 3:**
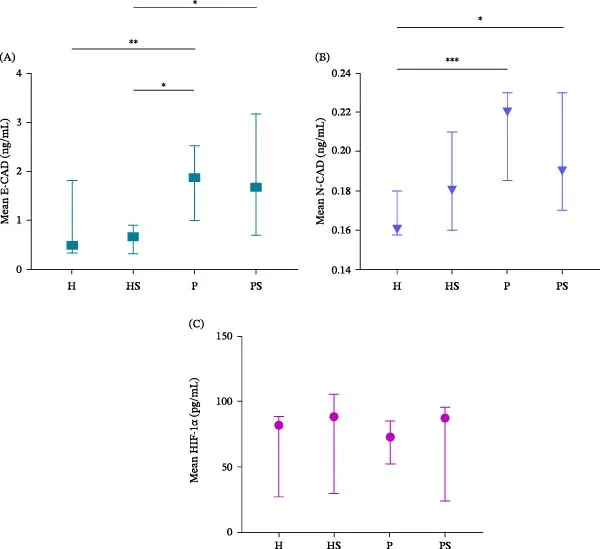
Salivary biomarker levels across the study groups. Comparison of salivary (A) E‐cadherin, (B) N‐cadherin, and (C) HIF‐1α levels among periodontally healthy nonsmokers (H), healthy smokers (HS), nonsmoking patients with periodontitis (P), and smokers with periodontitis (PS). Data are presented as mean values with error bars. Intergroup differences were assessed using the Kruskal–Wallis test followed by post hoc pairwise comparisons, significant at  ^∗^ *p* < 0.05,  ^∗∗^ *p*  < 0.01, and  ^∗∗∗^ *p* < 0.001.

The salivary concentrations of E‐CAD, N‐CAD, and HIF‐1α are presented in Table 2 and Figure 3. Significant differences in salivary E‐CAD and N‐CAD levels were observed among the study groups (*p*  < 0.001). Both biomarkers were significantly elevated in the periodontitis groups compared with the healthy groups. E‐CAD levels were highest in the periodontitis group (2.10 ± 1.18 ng/mL), followed by the smokers with periodontitis group (1.76 ± 1.49 ng/mL). Similarly, N‐CAD levels were higher in participants with periodontitis than in healthy participants. In contrast, salivary HIF‐1α levels did not differ significantly among the study groups (*p* = 0.272).

**Table 2:** Salivary biomarker levels across the study groups.

Parameters	H	HS	P	PS	*p*‐Value
E.CAD (ng/mL)					
Mean ± SD	0.920 ± 0.740	0.610 ± 0.330	2.100 ± 1.180	1.760 ± 1.490	<0.001 ^∗∗∗^
Median	0.500	0.660	2.090	1.360	
Min	0.010	0.010	0.050	0.001	
Max	2.300	1.130	4.710	4.731	
N.CAD (ng/mL)					
Mean ± SD	0.170 ± 0.030	0.192 ± 0.042	0.21 ± 0.03	0.200 ± 0.004	<0.001 ^∗∗∗^
Median	0.182	0.180	0.220	0.160	
Min	0.132	0.150	0.150	0.150	
Max	0.282	0.280	0.290	0.280	
HIF‐1α (pg/mL)					
Mean ± SD	69.970 ± 32.00	81.510 ± 48.24	69.000 ± 29.920	73.55 ± 45.57	0.272^**NS**^
Median	81.750	88.250	72.500	87.000	
Min	19.380	0.460	16.880	19.250	
Max	118.500	185.00	115.00	189.750	

*Note:* Comparison was performed using the Kruskal–Wallis test.

Abbreviations: E‐CAD, E‐cadherin; H, healthy nonsmokers; HIF‐1α, hypoxia‐inducible factor‐1 alpha; HS, healthy smokers; Max, maximum; Min, minimum; N‐CAD, N‐cadherin; NS, nonsignificant; P, nonsmokers with periodontitis; PS, periodontitis smoker; SD, standard deviation.

^∗∗∗^ *p*  < 0.001 statistically significant.

### 3.4. Correlation Analysis

Correlation analysis demonstrated predominantly weak or nonsignificant associations between salivary biomarkers and clinical periodontal parameters (Table 3). A weak positive correlation was observed between N‐CAD and PPD in the healthy group (*r* = 0.311, *p* = 0.041), while a moderate positive correlation was identified between N‐CAD and CAL in the periodontitis group (*r* = 0.411, *p* = 0.046). No significant correlations were found between E‐CAD and HIF‐1α and the examined clinical periodontal parameters. Interbiomarker analysis revealed a moderate positive correlation between N‐CAD and HIF‐1α in the periodontitis group (*r* = 0.45, *p* = 0.020). In contrast, E‐CAD was negatively correlated with N‐CAD in the smokers with periodontitis group (*r* = −0.48, *p* = 0.010) (Table 4).

**Table 3:** Correlations between salivary biomarkers and clinical periodontal parameters within each study group.

Groups		E‐CAD (ng/mL)		N‐CAD (ng/mL)		HIF‐1α (pg/mL)	
		*r*	*p*‐Value	*r*	*p*‐Value	*r*	*p*‐Value
H	PI (%)	0.160	0.445	−0.091	0.669	0.041	0.831
	BOP (%)	−0.050	0.783	0.211	0.314	0.161	0.451
	PPD (mm)	−0.140	0.343	0.311	0.041 ^∗^	0.021	0.851
	CAL (mm)	0.080	0.573	−0.031	0.087	0.240	0.101
HS	PI (%)	0.070	0.712	0.351	0.088	0.130	0.531
	BOP (%)	−0.040	0.841	0.051	0.796	−0.040	0.851
	PPD (mm)	−0.100	0.641	0.021	0.992	−0.160	0.451
	CAL (mm)	−0.070	0.721	−0.151	0.479	0.090	0.671
P	PI (%)	0.070	0.741	0.262	0.214	0.140	0.511
	BOP (%)	0.040	0.841	0.212	0.309	0.040	0.841
	PPD (mm)	−0.090	0.671	0.192	0.368	−0.040	0.8312
	CAL (mm)	−0.320	0.111	0.411	0.046 ^∗^	0.120	0.562
PS	PI (%)	−0.222	0.291	0.311	0.133	−0.110	0.602
	BOP (%)	−0.011	0.991	0.021	0.916	0.110	0.592
	PPD (mm)	0.273	0.191	−0.032	0.856	0.010	0.952
	CAL (mm)	−0.053	0.791	0.381	0.066	0.250	0.212

Abbreviations: BOP, bleeding on probing; CAL, clinical attachment loss; E‐CAD, E‐cadherin; H, healthy nonsmokers; HIF‐1α, hypoxia‐inducible factor‐1 alpha; HS, healthy smokers; N‐CAD, N‐cadherin; P, nonsmokers with periodontitis; PI, plaque index; PPD, probing pocket depth; PS, periodontitis smokers; *r*, Spearman correlation coefficient.

^∗^ *p*  < 0.05 statistically significant.

**Table 4:** Correlations among salivary E‐cadherin, N‐cadherin, and HIF‐1α within each study group.

Interaction		E‐CAD (ng/mL)		N‐CAD (ng/mL)	
		*r*	*p*‐Value	*r*	*p*‐Value
H	HIF‐1a (pg/mL)	0.010	0.940	0.030	0.860
HS	HIF‐1a (pg/mL)	0.010	0.930	0.120	0.560
P	HIF‐1a (pg/mL)	0.030	0.870	0.450	0.020^∗^
PS	HIF‐1a (pg/mL)	0.140	0.480	−0.100	0.630
H	N‐CAD (ng/mL)	0.010	0.930	—	—
HS	N‐CAD (ng/mL)	0.120	0.560	—	—
P	N‐CAD (ng/mL)	0.160	0.430	—	—
PS	N‐CAD (ng/mL)	−0.480	0.010^∗^	—	—

Abbreviations: E‐CAD, E‐cadherin; H, healthy nonsmokers; HIF‐1α, hypoxia‐inducible factor‐1 alpha; HS, healthy smokers; N‐CAD, N‐cadherin; p, nonsmokers with periodontitis; PS, periodontitis smokers; *r*, Spearman correlation coefficient.

^∗^ *p*  < 0.05 statistically significant.

### 3.5. Diagnostic Performance of Salivary Biomarkers

**Figure 4:**
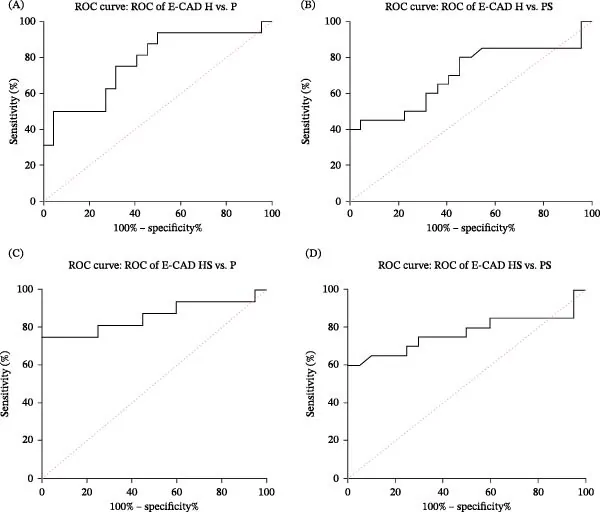
Receiver operating characteristic curves of salivary E‐cadherin. (A) Salivary concentration of E‐cadherin shows diagnostic potential to differentiate periodontal health from periodontitis (AUC 77.2%). (B) Salivary E‐cadherin exhibits diagnostic potential to differentiate health from periodontitis smokers (AUC 70.1%). (C) In addition, good diagnostic ability of this biomarker to discriminate between healthy smokers from periodontitis (AUC 85.9%) was observed. (D) Salivary E‐cadherin exhibits diagnostic potential to differentiate healthy smokers from periodontitis smokers (AUC 77.1%).

**Figure 5:**
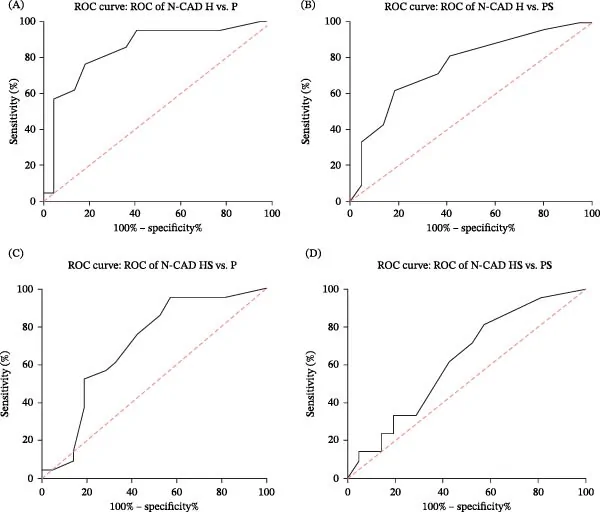
Receiver operating characteristic curves of salivary N‐cadherin. (A) Salivary concentration of N‐cadherin shows good diagnostic potential to differentiate periodontal health from periodontitis (AUC 84.5%). (B) Salivary N‐cadherin exhibits diagnostic potential to differentiate health from periodontitis smokers (AUC 75.9%). (C) In addition, fair diagnostic ability of this biomarker to discriminate between healthy smokers and periodontitis (AUC 70%) was observed. (D) Salivary N‐cadherin exhibits poor diagnostic potential to differentiate healthy smokers from periodontitis smokers (AUC 62.2%).

ROC analysis demonstrated acceptable discriminatory performance for both N‐CAD and E‐CAD across several group comparisons. N‐CAD showed the highest discrimination between healthy participants and those with periodontitis (AUC = 84.5%). It also demonstrated fair discriminatory performance for distinguishing healthy participants from smokers with periodontitis (AUC = 75.9%) and healthy smokers from participants with periodontitis (AUC = 70.0%). For E‐CAD, the highest discriminatory performance was observed in distinguishing healthy smokers from participants with periodontitis (AUC = 85.9%). Fair discriminatory performance was also observed for differentiating healthy participants from those with periodontitis (AUC = 77.2%) and smokers with periodontitis (AUC = 70.1%). In addition, E‐CAD showed fair discriminatory ability in distinguishing healthy smokers from smokers with periodontitis (AUC = 77.1%). In contrast, HIF‐1α showed variable discriminatory performance across the evaluated comparisons, with no consistent pattern of discrimination between periodontal health and disease (Table 5 and Figures 4 and 5).

**Table 5:** Diagnostic performance of salivary biomarkers.

Groups	Biomarkers	AUC	95% CI	*p*‐Value	Optimal cutoff point	% Sensitivity		% Specificity
H vs. HS	E‐CAD (ng/mL)	57.3%	0.394–0.753	0.413	1.140	100.000		36.360
	N‐CAD (ng/mL)	61.9%	0.448–0.789	0.181	0.185	42.860		81.820
	HIF‐1α (pg/mL)	74.7%	0.593–0.901	0.006^∗^	86.880	60.000		86.360
H vs. P	E‐CAD (ng/mL)	77.2%	0.617–0.928	0.005^∗^	1.985	50.000		95.450
	N‐CAD (ng/mL)	84.5%	0.721–0.968	<0.001 ^∗^	0.165	76.190		81.900
	HIF‐1α (pg/mL)	53.3%	0.356–0.711	0.706	51.630	80.950		40.910
H vs. PS	E‐CAD (ng/mL)	70.1%	0.535–0.867	0.026^∗^	1.960	45.000		95.450
	N‐CAD (ng/mL)	75.9%	0.614–0.905	0.004	0.185	61.900		84.440
	HIF‐1α (pg/mL)	89.8%	0.804–0.991	<0.001 ^∗^	86.130	84.210		81.820
HS vs. P	E‐CAD (ng/mL)	85.9%	0.718–1.000	<0.001 ^∗^	1.250	75.000		100.000
	N‐CAD (ng/mL)	70.0%	0.536–0.864	0.026^∗^	0.165	95.240		42.860
	HIF‐1α (pg/mL)	67.3%	0.504–0.843	0.057	86.00	80.950		60.000
HS vs. PS	E‐CAD (ng/mL)	77.1%	0.611–0.931	0.003	1.160	60.000		100.000
	N‐CAD (ng/mL)	62.2%	0.451–0.793	0.174	0.165	80.950		47.620
	HIF‐1α (pg/mL)	61.9%	0.441–0.798	0.201	80.130	89.470		40.000
P vs. PS	E‐CAD (ng/mL)	52.5%	0.333–0.716	0.799	1.280	45.000		75.000
	N‐CAD (ng/mL)	59.5%	0.418–0.771	0.291	0.205	66.670		57.140
	HIF‐1α (pg/mL)	83.2%	0.701–0.963	<0.001 ^∗^	85.75	84.21		80.950

Abbreviations: AUC, area under the curve; CI, confidence interval.

^∗^Significance level at *p*  < 0.05.

### 3.6. Mixed‐Effects Model Analysis

Mixed‐effects modeling was performed to evaluate the independent associations of smoking and periodontitis with biomarker levels and clinical periodontal parameters. Periodontitis was independently associated with higher E‐CAD levels (β = 1.49, *p* = 0.002) and increased periodontal parameters (*p*  < 0.001). Similarly, smoking was independently associated with higher E‐CAD levels (β = 1.15, *p* = 0.017) and increased periodontal parameters (*p*  < 0.001). In contrast, neither smoking nor periodontitis showed significant associations with N‐CAD or HIF‐1α levels (*p*  > 0.05) (Table 6).

**Table 6:** Mixed‐effects model estimates of smoking and periodontitis effects on biomarkers and periodontal parameters.

Biomarker	Effect	Estimate	Std. error	*t*‐Value	*p*‐Value
E‐CAD (ng/mL)	Intercept (H smoker)	0.610	0.070	8.340	0.001 ^∗^
	Health effect	0.310	0.170	1.760	0.153
	P smoker effect	1.150	0.340	3.360	0.017^∗^
	Periodontitis effect	1.490	0.300	4.890	0.002^∗^
N‐CAD (ng/mL)	Intercept (H smoker)	0.190	0.010	23.04	0.001^∗^
	Health effect	−0.020	0.010	−1.570	0.195
	P smoker effect	0.010	0.010	1.100	0.348
	Periodontitis effect	0.020	0.010	1.900	0.128
HIF‐1a (pg/mL)	Intercept (H smoker)	81.530	10.790	7.560	0.001^∗^
	Health effect	−11.560	12.760	−0.910	0.434
	P smoker effect	−7.980	15.020	−0.530	0.641
	Periodontitis effect	−12.530	12.610	−0.990	0.393
PI%	Intercept (H smoker)	7.590	0.310	24.250	0.001^∗^
	Health effect	0.080	0.500	0.160	0.884
	P smoker effect	59.840	3.960	15.090	0.001^∗^
	Periodontitis effect	58.670	3.480	16.870	0.001^∗^
BOP%	Intercept (H smoker)	4.170	0.380	10.840	0.001^∗^
	Health effect	−0.460	0.620	−0.750	0.513
	P smoker effect	38.850	2.460	15.780	*0.001* ^∗^
	Periodontitis effect	46.840	5.160	9.070	0.001^∗^
PPD (mm)	Intercept (H smoker)	1.820	0.180	10.000	0.001^∗^
	Health effect	0.050	0.250	0.190	0.869
	P smoker effect	2.800	0.190	14.590	0.001^∗^
	Periodontitis effect	2.710	0.200	13.280	0.001^∗^
CAL (mm)	Intercept (H smoker)	1.500	0.130	11.620	0.001^∗^
	Health effect	−0.100	0.180	−0.540	0.633
	P smoker effect	1.310	0.180	7.230	0.001^∗^
	Periodontitis effect	1.280	0.190	6.640	0.001^∗^

Abbreviations: BOP, bleeding on probing; CAL, clinical attachment loss; Data are presented as regression coefficient (Estimate), standard error (SE), t‐value, and *p*‐value. The intercept represents the reference group (healthy smokers); E‐CAD, E, cadherin; HIF‐1 α: Hypoxia‐inducible factor‐1alpha; N‐CAD, N, cadherin; PI, plaque index; PPD, probing pocket depth.

^∗^Indicates statistical significance at *p* < 0.05.

## 4. Discussion

The present study evaluated the impact of smoking and periodontitis on salivary biomarkers E‐CAD, N‐CAD, and HIF‐1α levels and examined their relationship with clinical periodontal parameters. Participants with periodontitis exhibited significantly higher clinical periodontal measurements and alterations in selected salivary biomarkers compared with periodontally healthy controls.

The clinical periodontal parameters PI, BOP, PPD, and CAL were significantly higher in periodontitis groups compared with healthy groups. This finding is consistent with the established pathophysiology of periodontitis, where bacterial biofilm induces a chronic inflammatory response, leading to tissue destruction and attachment loss [34, 35]. The lack of significant differences in clinical periodontal parameters between healthy smokers and healthy nonsmokers suggests that smoking alone may not produce readily detectable clinical changes in the absence of established periodontitis. Nevertheless, smoking‐related vascular, inflammatory, and molecular alterations may occur before becoming clinically apparent [11, 36]. Continual exposure of the periodontal epithelium to microbial biofilms, inflammatory cytokines, and growth factors may facilitate epithelial remodeling and alterations associated with EMT [9, 37]. Changes in the expression of biomarkers associated with EMT are acknowledged characteristics of this process. Nonetheless, EMT cannot be determined only by changes in genetic markers, as it also encompasses coordinated morphological, functional, and behavioral modifications in epithelial cells [38].

Regarding salivary biomarkers, E‐CAD levels were significantly higher in the periodontitis groups than in periodontally healthy individuals. E‐CAD is essential for maintaining epithelial integrity, as it mediates intercellular adhesion and serves as a primary determinant of the epithelial phenotype [39]. The observed increase in soluble E‐CAD may initially appear contradictory, as E‐CAD expression is generally downregulated in the tissue context during EMT [40, 41]. However, this finding may reflect disruption of the periodontal epithelial barrier rather than increased cellular expression of E‐CAD. Membrane‐bound E‐CAD can be cleaved by proteolytic enzymes, particularly matrix metalloproteinases, potentially leading to the liberation of soluble E‐CAD fragments into saliva [42]. Moreover, proteases from periodontopathogens, specifically *Porphyromonas gingivalis*, can cross the epithelial barrier and penetrate the underlying connective tissue through a paracellular route by degrading junctional proteins [43]. Consistent with the present findings, Kazem et al. [44] posited that the elevated salivary free E‐CAD levels in periodontitis may reflect inflammatory tissue destruction and the subsequent release of adhesion molecules into oral fluids. Together, these observations suggest that increased salivary E‐CAD may be associated with inflammation‐related disruption of the periodontal epithelium. Therefore, elevated salivary E‐CAD levels are more likely to reflect proteolytic shedding and inflammation‐related disruption of the periodontal epithelium associated with periodontitis.

Similarly, N‐CAD levels were significantly higher in the periodontitis groups than in the periodontally healthy groups. As N‐CAD is typically associated with mesenchymal phenotype, its upregulation may reflect epithelial remodeling and possible EMT‐related alteration in periodontal tissues [9, 45]. Previous studies have described a “cadherin switch,” characterized by reduced E‐CAD tissue expression accompanied by increased N‐CAD expression, which is associated with weakened intercellular adhesion and altered cellular behavior [46–48]. Consistent with the findings of Alanbari et al. [37], the combined alterations in E‐CAD and N‐CAD observed in the present study may reflect epithelial remodeling and possible EMT‐related alterations in periodontal tissues. However, the concurrent elevation of both biomarkers in saliva represents an apparent paradox that underscores the distinction between tissue expression and soluble biomarker release into saliva.

Smoking appears to modify biomarker expression, particularly E‐CAD, through several mechanisms. Although cigarette smoke has been reported to disrupt epithelial integrity and downregulate tissue E‐CAD expression, salivary E‐CAD reflects the soluble extracellular fraction released into saliva rather than membrane‐bound cellular expression [49]. Inflammatory tissue injury and proteolytic cleavage may enhance the release of E‐CAD in saliva, whereas smoking‐related alteration in gingival blood flow and gingival crevicular fluid (GCF) dynamics may influence biomarker detectability in salivary samples [11, 50]. Accordingly, the slightly lower salivary E‐CAD concentration observed in smokers with periodontitis should be interpreted cautiously, particularly as post hoc analysis revealed no statistically significant differences between periodontitis smokers and nonsmokers for most biomarkers and clinical parameters. In contrast, the mixed‐effects model showed that smoking was independently associated with higher E‐CAD levels, suggesting that smoking‐related effects may be more evident after accounting for multiple study variables. Supporting our observations, Güney et al. reported that smoking is associated with decreased levels of tissue‐derived biomarkers in GCF [51].

Hypoxia is frequently observed in chronically inflamed tissues and has been implicated in several vascular and inflammatory processes [20]. Although previous studies have suggested a potential association between HIF‐1α signaling and EMT‐related pathways, the present study found no statistically significant differences in salivary HIF‐1α levels among the study groups [25]. Several reasons may explain this finding. HIF‐1α is an intracellular molecule that rapidly degrades under normal oxygen tension conditions, limiting its stability and detectability in saliva [52, 53]. In addition, dilution in whole saliva may mask subtle fluctuations in biomarker concentrations. Smoking‐induced systemic hypoxia may contribute in baseline elevation of HIF‐1α levels among smokers reducing intergroup variance. The relatively wide variability observed among smokers may have further limited the ability to detect group differences.

HIF‐1α was not consistently associated with EMT‐related biomarkers, except for an isolated positive correlation with N‐CAD in the periodontitis group. This isolated finding should be interpreted cautiously and may reflect a group‐specific relationship rather than a consistent association across the study population. Collectively, these findings suggest that salivary HIF‐1α may be influenced by localized tissue conditions and has limited and inconsistent ability to reflect periodontal hypoxia or distinguish periodontal health from disease.

Previous studies have reported that hypoxia‐related biomarkers may be detected more reliably in site‐specific biological fluids, such as GCF and tissue samples, than in whole saliva [13, 18, 54, 55]. Afacan et al. [18] reported that salivary HIF‐1α levels may not consistently reflect periodontal status due to the heterogeneous nature of the saliva. Similarly, Bozyel et al. [13] observed that hypoxia‐associated alterations are more clearly identified in GCF than in saliva, emphasizing the importance of site‐specific fluid analysis. Taşdemir et al. also reported that salivary HIF‐1α concentration failed to distinguish between different disease severity in spite of an overall increase in salivary HIF‐1α in periodontitis [56]. In contrast, Kzar and Abbas [57] observed significantly elevated salivary HIF‐1α levels in participants with periodontitis. These inconsistencies may reflect differences in study design, participant characteristics, disease classification, sample collection and processing, and analytical methods.

Correlation analyses in the present study demonstrated predominantly weak or nonsignificant associations between salivary biomarkers and clinical parameters. Although a moderate correlation between N‐CAD and CAL in the periodontitis group, most correlations were weak, suggesting that no single biomarker adequately captures the complexity of periodontal disease. The findings suggest that although the examined biomarkers may correlate with disease presence, their levels do not necessarily align with all clinical measures, highlighting the multifactorial nature of periodontitis [35]. No significant associations were identified between salivary HIF‐1α and clinical periodontal parameters across the examined groups. In smokers, the absence of clear biomarker correlations may partly reflect smoking‐related changes in vascular function and immune responses, which could confound salivary biomarker variability and clinical presentation [58]. Similarly, despite the elevated salivary E‐CAD levels observed in periodontitis, no significant correlations with clinical parameters were detected, suggesting that salivary E‐CAD may reflect epithelial damage instead of cumulative disease severity.

Importantly, the mixed‐effects model showed that both smoking and periodontitis were independently associated with higher E‐CAD levels and worsened periodontal parameters. In contrast, no significant associations were observed for N‐CAD or HIF‐1α levels. These findings may indicate that E‐CAD may be a more sensitive biomarker for detecting the combined effects of smoking and periodontal disease. ROC analysis demonstrated acceptable discriminatory performance for E‐CAD and N‐CAD in distinguishing participants with periodontitis from periodontally healthy controls. However, the performance of N‐CAD should be interpreted cautiously because it was not significantly associated with smoking or periodontitis in the mixed‐effects model. In contrast, HIF‐1α showed inconsistent ROC performance. Consistent with the present findings, previous studies have reported that salivary E‐CAD may demonstrate potential utility in differentiating periodontitis from periodontal health status [44]. Although the sample size was adequate for the primary group comparisons, larger studies may provide greater precision for correlation, ROC, mixed‐effects, and subgroup analyses and enhance the generalizability of the findings.

Taken together, these findings suggest that salivary E‐CAD, and to a lesser extent N‐CAD, may serve as adjunctive minimally invasive approach indicators of epithelial disruption and tissue remodeling associated with periodontal disease. To reduce potential sources of bias in the research process, a predefined sample, calibrated methods, and blinding protocols for both the ELISA investigator and statistician were employed. Together, these measures enhance the reliability of the existing biomarker’s diagnostic efficacy. Periodontitis staging and grading were not incorporated because the primary objective was to evaluate salivary E‐CAD, N‐CAD, and HIF‐1α levels according to smoking status and the presence of periodontitis rather than disease severity or progression.

However, several limitations should be acknowledged. First, the case–control design restricts causal inference. Second, age was not included in the multivariable analysis and may represent a source of residual confounding, despite the absence of statistically significant age differences between groups. The unequal sex distribution among study groups, largely reflecting the limited availability of female cigarette smokers in the local population, means that sex‐related confounding cannot be completely excluded. The lack of detailed quantitative smoking‐exposure data, particularly pack‐years and individual daily cigarette consumption, limited the assessment of dose–response relationships between tobacco exposure, periodontal clinical parameters, and salivary biomarker levels.

Future research should focus on longitudinal designs to monitor dynamic changes in biomarker levels during treatment and follow‐up. Periodontitis stage and grade, cumulative smoking exposure, salivary flow rate, and total salivary protein should also be considered. Variations in salivary flow and composition may influence oral biofilm accumulation, inflammatory status, and biomarker concentrations [59]. Furthermore, the simultaneous analysis of saliva, GCF, and periodontal tissues may further clarify the relationship between localized hypoxia, EMT‐related changes, and salivary biomarker concentrations.

## 5. Conclusion

Salivary E‐CAD and N‐CAD levels were significantly altered in individuals with periodontitis, supporting their potential as noninvasive biomarkers associated with periodontal disease. The observed elevations in salivary E‐CAD may reflect epithelial disruption and proteolytic shedding, while the combined changes in E‐CAD and N‐CAD are consistent with EMT‐related alterations reported in periodontal inflammation. In contrast, salivary HIF‐1α showed limited discriminatory ability and was not consistently associated with periodontal status or clinical parameters. Smoking was independently associated with higher E‐CAD levels, although its effect was less pronounced than that of periodontitis. Overall, EMT‐associated biomarkers demonstrated greater potential than HIF‐1α for reflecting periodontal disease status in saliva. Future longitudinal studies integrating salivary, tissue, and GCF analyses are recommended to better elucidate the interplay between hypoxia and EMT‐associated biomarkers in periodontal disease progression.

## Author Contributions

**Ban Emad Kareem**: data curation, validation, methodology, writing – original draft, writing – review and editing, conceptualization. **Saif Sehaam Saliem**: project administration, writing – original draft, supervision, methodology, investigation, conceptualization, writing – review and editing.

## Funding

This study did not receive any specific funding.

## Disclosure

All authors have read and approved the manuscript. After using Quillbot Premium, Ban Emad Kareem reviewed and edited the content as needed and took full responsibility for the content of the publication.

## Ethics Statement

The research received ethical approval from the Ethics Committee of Clinical Research of the College of Dentistry, University of Baghdad (Approval Number 1119; 27 November 2025).

## Conflicts of Interest

The authors declare no conflicts of interest.

## Data Availability

The data that support the findings of this study are available from the corresponding author upon reasonable request.
